# Filariasis of Stensen's Duct: An Index Case

**DOI:** 10.1155/2016/7646451

**Published:** 2016-10-27

**Authors:** Eishaan K. Bhargava, Nikhil Arora, Varun Rai, Ravi Meher, Prerna Arora, Ruchika Juneja

**Affiliations:** ^1^Department of Ent and Head and Neck Surgery, Maulana Azad Medical College, New Delhi, India; ^2^Department of Pathology, Maulana Azad Medical College, New Delhi, India

## Abstract

Filariasis, a neglected tropical disease, is a global health problem and is endemic to 73 countries including India. It is caused by nematodes of Filariodidea family, namely,* W. bancrofti* and* B. malayi* in India, which have a predilection for the lower limbs and testis. We report a never before reported case of filariasis of the main parotid duct in a 25-year-old male that resolved on medical management, exemplifying the importance of maintaining a high index of suspicion and careful examination of cytological smears in endemic countries, allowing for an early diagnosis and treatment, decreasing the morbidity of this debilitating disease.

## 1. Introduction

Filariasis, a neglected tropical disease, is a global health problem affecting 73 countries, including India, with a population of over 1.2 billion at risk for infection [1]. It is caused by infection with nematodes of the family Filariodidea, namely,* Wuchereria bancrofti* (90% cases),* Brugia malayi* (majority of remainder cases), and* B. timori* (rare), of which the former two are found in India. Although these parasites have a marked predilection for lower limb lymphatics, the epididymis, and the spermatic cord [2], they have also been reported to occur at unusual sites such as the thyroid gland [3], body fluids [4], skin [5], breast [6], and the oral or perioral region [7]. Salivary gland involvement is very rare and has been reported only once previously [8]. Here, we report a previously unreported case of filariasis of the parotid duct in a young adult male.

## 2. Case Report

A 25-year-old man, resident of Uttar Pradesh, presented to the otorhinolaryngology outpatient department with a painless swelling of the left cheek, gradually increasing in size since he first noticed it 1 year earlier. He gave a history of temporary increase in size of the swelling while eating, with subsequent return to previous size on completion of a meal. On examination, a 2 cm × 1.5 cm globular swelling was present just below the left malar prominence, which, on palpation, was nontender, euthermic, firm, and mobile in all directions with no fixity to skin or underlying tissues and a grossly normal overlying skin. The swelling increased in prominence when the patient was made to clench his teeth. He was afebrile, with no lymphadenopathy or organomegaly. His complete blood counts revealed absolute eosinophilia (1100/mm^3^), with an essentially normal peripheral smear.

**Figure 1:**
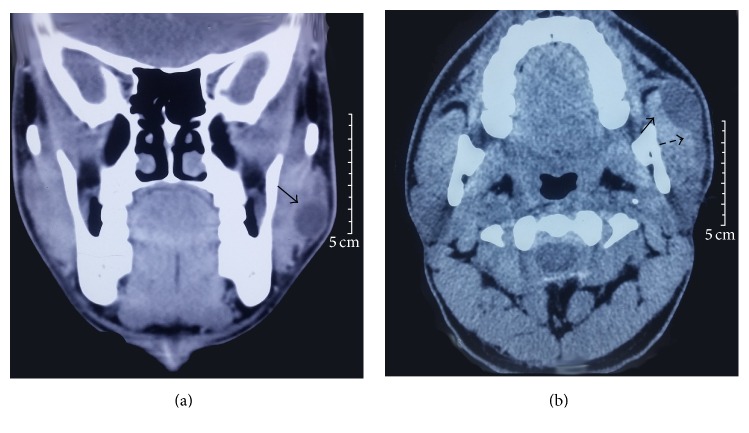
Coronal (a) and axial (b) cuts of computed tomography scan showing a small 2.2 cm × 1.5 cm, well defined, hypodense cystic lesion superficial to the anterolateral aspect of the left masseter muscle indenting its surface (solid arrow), communicating with a tubular hypodense structure (dashed arrow) communicating with the parotid gland, suggestive of a dilated Stensen's duct with a sialocele formation.

On ultrasonography, a cystic dilatation of the middle part of the left Stensen's duct was seen with minimal lobulated soft tissue contents and a mildly thickened duct wall. A contrast enhanced computed tomography scan was done that showed a small, well defined, hypodense cystic lesion superficial to the anterolateral aspect of the left masseter muscle indenting its surface, communicating with a tubular hypodense structure communicating with the parotid gland, suggestive of a dilated Stensen's duct with a sialocele formation (Figures 1(a) and 1(b)).

After clinical and radiological examination, the differential diagnosis that came to our mind was that it is either a sialolith blocking the duct that has lead to the inflammatory swelling or cysticercosis but further investigations showed a diagnosis which was very different and rare.

**Figure 2:**
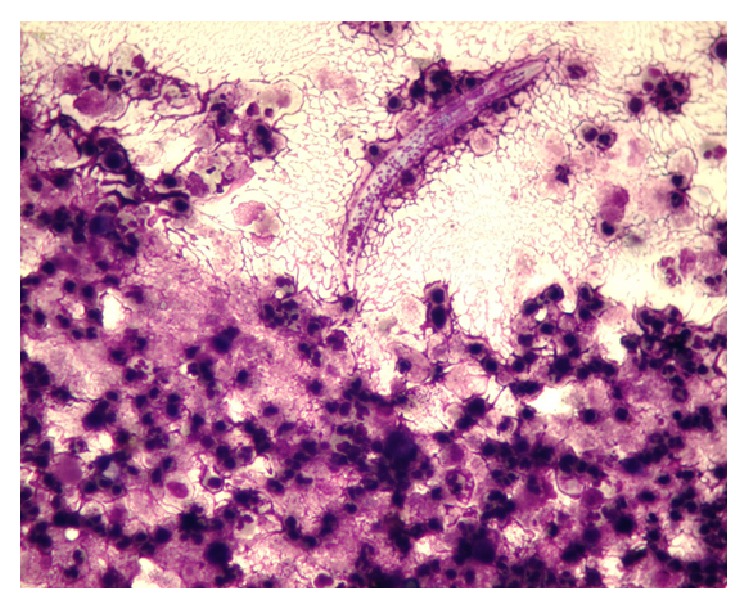
Fine needle aspiration cytology showing macrophages and nucleated squamous cells in a dense acute suppurative background with degenerated microfilariae.

Fine needle aspiration cytology (FNAC) revealed macrophages and nucleated squamous cells in a dense acute suppurative background with degenerated microfilariae, with no acid-fast bacilli (Figure 2).

On the basis of the radiological and cytological findings, a final diagnosis of filariasis of the parotid duct was made, and the patient was started on a two-week course of diethyl carbamazine, along with a five-day adjunctive course of albendazole.

On completion of the medical management, the patients symptoms were completely resolved, and he remains asymptomatic after 10 months of regular follow-up.

## 3. Discussion

Filariasis is a disfiguring and debilitating disease endemic to 73 nations worldwide, including India, with an estimated 600 million people at risk in 250 endemic districts of India, with the highest burden of disease in the states of Uttar Pradesh, Bihar, Jharkhand, Andhra Pradesh, Kerala, and Gujarat [9]. The causative organisms in India are mainly* W. bancrofti* and* B. malayi*, with subcutaneous filariasis caused by* Loa loa*,* Onchocerca volvulus*, and* Mansonella* [7].

Clinically apparent infection occurs only in a small proportion of infected individuals and may be classified either as* lymphatic filariasis*, caused by the presence of the parasite in the lymphatic system, or* occult filariasis*, due to an immune hyperresponsiveness of the host towards the parasite. In lymphatic filariasis, microfilariae may be found circulating in the bloodstream and may present in the acute phase as filarial fever, lymphangitis, lymphadenitis, and lymphedema. This would progress to the chronic phase over years, leading to permanent structural changes. Occult or cryptic filariasis refers to cases like ours where classical clinical signs of filariasis are absent with no circulating microfilariae. It is believed to be due to a hypersensitivity reaction to filarial antigens derived from microfilariae [8].

In the past, diagnosis of filariasis rested solely upon the demonstration of microfilariae in blood, making the definitive diagnosis tedious since blood samples would have to be taken at night between 2200 and 0400 hours [7].

FNAC has emerged as a useful diagnostic tool, used for the confirmatory diagnosis of suspected cases of filariasis, especially in cryptic cases where circulating microfilariae are absent, affecting the testis, epididymis, thyroid, breast, and subcutaneous nodules [7, 10]. The typical cytological picture comprises the detection of an adult worm or microfilarial form in a background of eosinophils, mononuclear cells, and neutrophils. It is usual to find associated epithelioid cell granulomas and giant cells, resulting in a diagnostic conundrum where mycobacterial infection needs to be ruled out using 20 and 5% Ziehl-Neelsen staining [7].

Filariasis may present in an unusual fashion in many different sites, ranging from subcutaneous nodules and thyroid gland filariasis to extremely rare cases like ours where salivary glands or their ducts are involved. This wide spectrum of unusual presentation necessitates a high index of suspicion and careful examination of cytological smears in endemic countries like ours. This rings true especially in this era of elimination of lymphatic filariasis (ELF), where the world is making extraordinary efforts to eliminate, and eventually eradicate, this disfiguring and debilitating disease that has been the cause for great mental, social, and economic loss to patients, contributing to stigma and poverty in the affected nations, including India. A timely diagnosis and management, as was done in the reported case, can not only alleviate one patient's suffering but also prevent the transmission of infection to many others, a small but important step towards the final goal of ELF.
